# Complications of Encrusted Stents in Recurrent Nephrolithiasis: A Case Report

**DOI:** 10.7759/cureus.82679

**Published:** 2025-04-21

**Authors:** Salim Lachkar, Ahmed Ibrahimi, Imad Boualaoui, Hachem El Sayegh, Yassine Nouini

**Affiliations:** 1 Urology A, Ibn Sina University Hospital, Rabat, MAR

**Keywords:** chronic kidney disease, encrusted stent, nephrolithiasis, nephrolithotomy, recurrent stones, renal obstruction, ureteral stent complications

## Abstract

Chronic kidney disease (CKD) and recurrent nephrolithiasis can lead to significant renal impairment due to obstruction, infections, and direct damage from stones. Timely intervention is crucial to prevent further deterioration, especially in patients with poorly controlled diabetes and encrusted stents. We present a 56-year-old male with poorly controlled type 2 diabetes and stage 3 CKD. He had recurrent nephrolithiasis and initially underwent ureteral stenting, followed by a second stent placement due to pyelonephritis and significant encrustation. Imaging revealed a large bilateral calculous mass. The patient initially underwent nephrolithotomy with spectrophotometric analysis of the stones and had a favorable postoperative outcome. This case highlights the impact of untreated stents and recurrent stones in CKD patients. Encrusted stents can cause hydronephrosis, secondary obstruction, and increased renal pressure, contributing to renal function decline. Surgical intervention, including nephrolithotomy and ureterolithotomy, can improve renal function and prevent further complications. Spectrophotometric analysis of the stones revealed struvite and uric acid stones, both linked to infections and metabolic factors. Early stone removal and management of encrusted stents are essential in CKD patients with recurrent nephrolithiasis. A multidisciplinary approach is necessary to optimize treatment and prevent further renal deterioration. Regular follow-up is critical to avoid irreversible renal damage.

## Introduction

Chronic kidney disease (CKD) and recurrent nephrolithiasis are closely linked conditions that can result in significant renal impairment due to obstructive episodes, infections, and direct stone-induced damage [1]. These complications are particularly concerning in patients with comorbidities, such as poorly controlled diabetes, where the risk of further renal deterioration is heightened [2]. In such patients, the use of ureteral stents is often necessary to manage obstructions; however, encrusted stents pose a significant challenge. The accumulation of mineral deposits on stents can exacerbate renal dysfunction and increase the risk of further complications, including hydronephrosis and infection [3]. Early intervention and careful management are critical to preventing long-term renal damage [4]. This case report highlights the clinical challenges and management strategies for a patient with encrusted stents, recurrent nephrolithiasis, and stage 3 CKD.

## Case presentation

We present the case of a 56-year-old male with a history of poorly controlled type 2 diabetes mellitus, stage 3 CKD, and recurrent nephrolithiasis. Due to worsening renal function with obstructive acute-on-chronic kidney injury, he previously underwent left ureteral stenting. However, he was subsequently lost to follow-up. Ten months later, a second double-J ureteral stent was inserted in parallel on the left side due to an episode of acute pyelonephritis. The initial stent, which had remained in place for 10 months, showed significant encrustation and could not be extracted despite multiple endoscopic attempts.

The patient presented to our department one month after the insertion of the second double-J stent, following the successful treatment and resolution of acute pyelonephritis, for the management of his significant lithiasis burden. Clinically, he had preserved diuresis but complained of chronic flank pain and intermittent macroscopic hematuria. Laboratory tests revealed impaired renal function with a serum creatinine level of 2.5 mg/dL (normal: 0.6-1.2 mg/dL), an estimated glomerular filtration rate (GFR) of 32 mL/min/1.73m^2^ (normal: >90 mL/min/1.73m^2^), and a blood urea nitrogen level of 55 mg/dL (normal: 7-20 mg/dL), consistent with stage 3 CKD. Inflammatory markers were within normal limits, with a C-reactive protein level of 8 mg/L (normal: <10 mg/L) and a white blood cell count of 8,500/mm^3^ (normal: 4,000-10,000/mm^3^). Urinalysis showed microscopic hematuria without pyuria, and urine culture, obtained before the initiation of antibiotic treatment, had isolated *Proteus mirabilis*, a urea-splitting organism commonly associated with infectious stones. The urine pH at that time was found to be 8.2, which is conducive to the formation of struvite stones (Table 1).

**Table 1:** Summary of laboratory results CKD: chronic kidney disease

Parameter	Result	Reference Range	Interpretation
Serum creatinine	2.5 mg/dL	0.6-1.2 mg/dL	Elevated (impaired renal function)
Estimated glomerular filtration rate	32 mL/min/1.73m^2^	>90 mL/min/1.73m^2^	Decreased (stage 3 CKD)
Blood urea nitrogen	55 mg/dL	7-20 mg/dL	Elevated (possible renal impairment)
C-reactive protein	8 mg/L	<10 mg/L	Normal
White blood cell count	8,500/mm^3^	4,000-10,000/mm^3^	Normal

**Figure 1:**
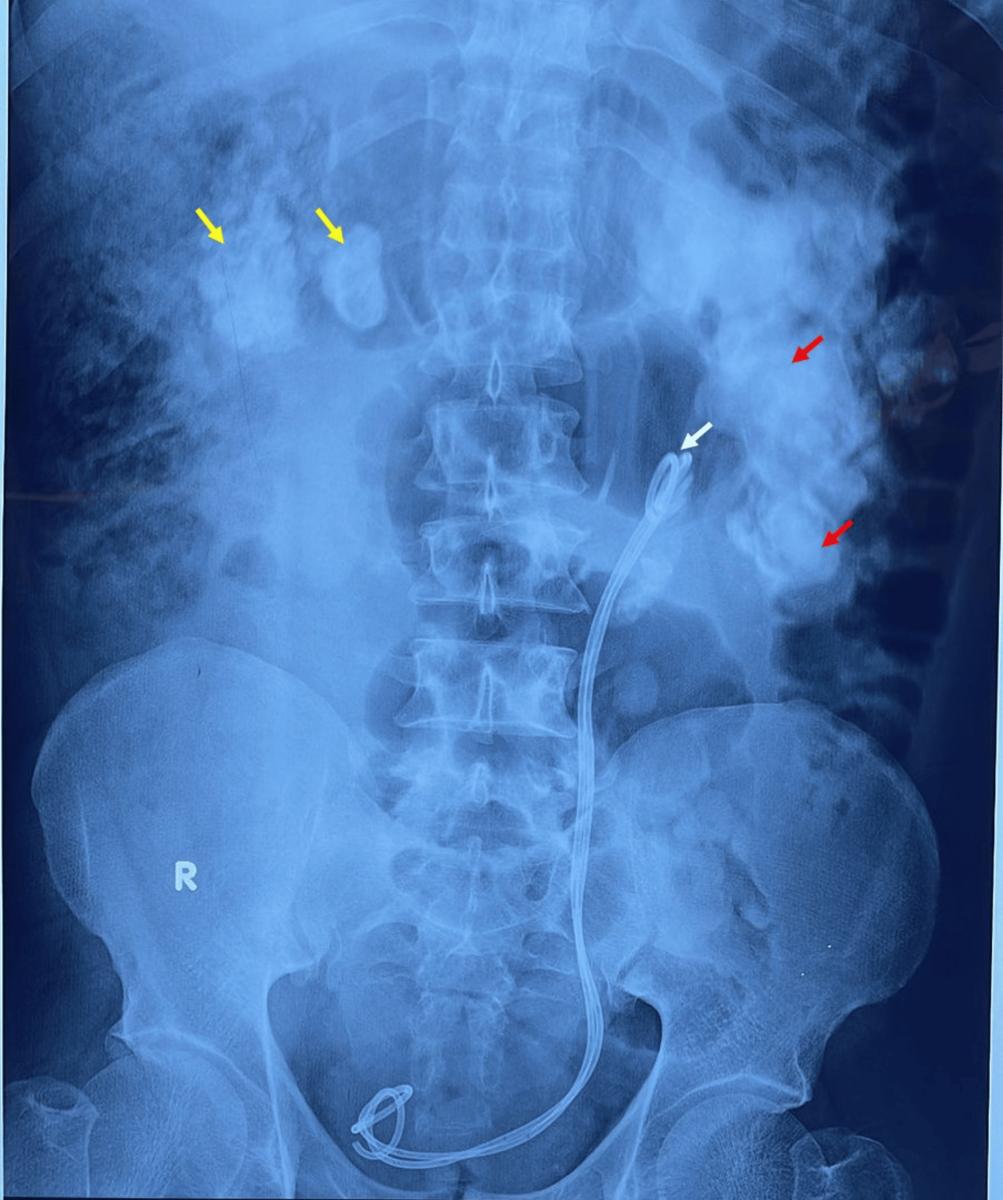
Abdominal plain X-ray showing bilateral radiopacities overlying the right (yellow arrows) and left (red arrows) renal areas. Two right-sided double-J stents are visible (white arrow).

An abdominal supine X-ray showed multiple radiopaque images in both renal fossae and along the left ureteral pathway. The encrusted double-J stent appeared as overlapping radiopaque lines with visible calcifications along their course, particularly at the ureterovesical junction. The second left double-J stent was seen in parallel (Figure 1).

**Figure 2:**
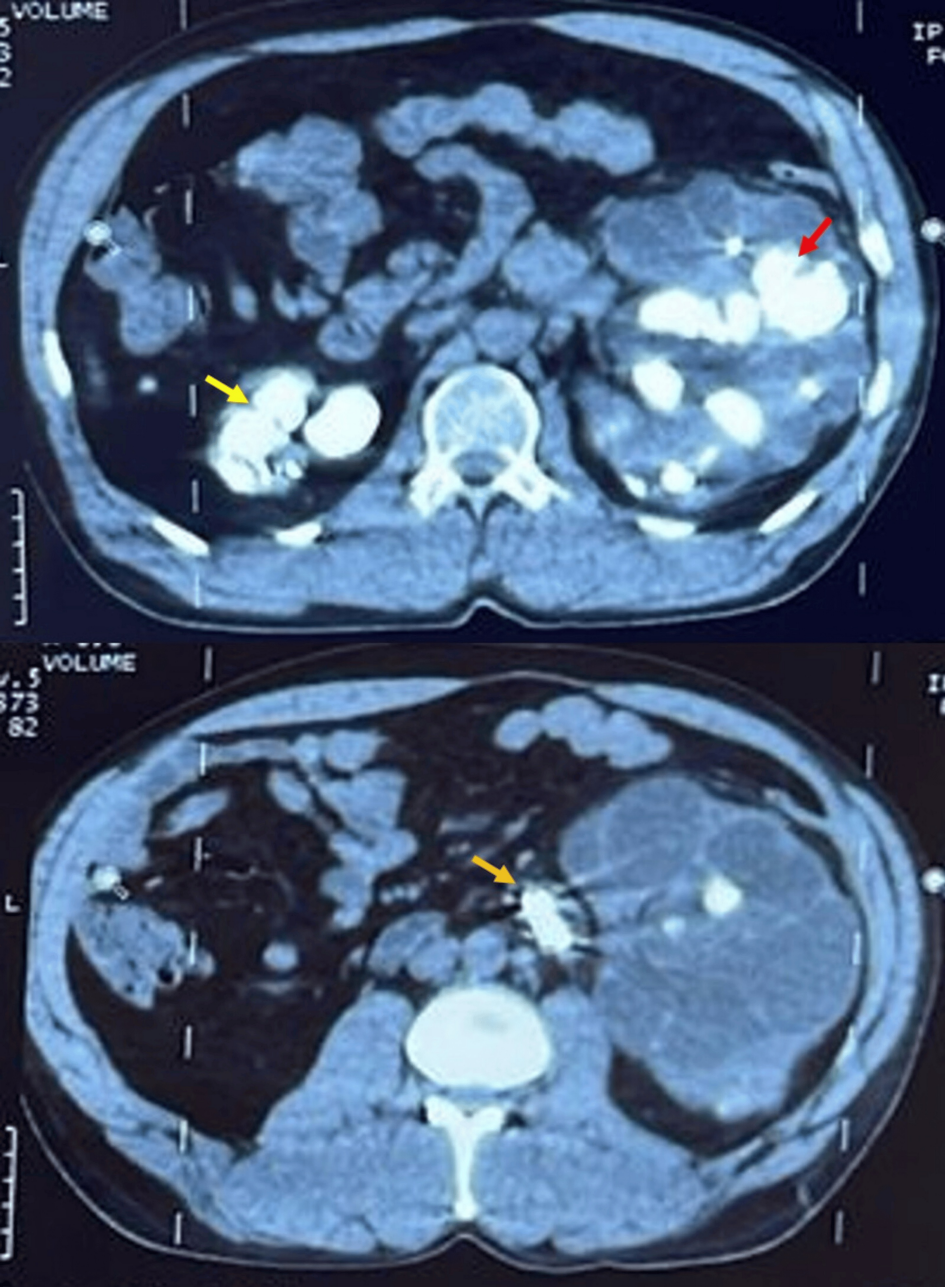
Non-contrast axial CT scan showing a small right kidney with multiple spontaneous hyperdensities corresponding to renal calculi (yellow arrow). The left kidney is hypertrophied as a compensatory mechanism and also presents multiple spontaneous hyperdensities (red arrow). A left ureteral calculus (orange arrow) is visible along the two double-J stents.

Imaging studies included a non-contrast CT scan, which revealed irregularly contoured kidneys with multiple large stones. The right kidney contained a lower calyceal stone measuring 33 × 32 mm with a density of 787 Hounsfield units and a pyelic stone measuring 30 × 20 mm with a density of 759 Hounsfield units. The left kidney had a significant lithiasis burden, with an upper calyceal stone extending into the renal pelvis, measuring 60 × 55 x 49 mm with a density of 791 Hounsfield units, and a mid-calyceal stone also extending into the renal pelvis, measuring 45x 33 × 32 mm with a density of 787 Hounsfield units. Additionally, three left ureteral stones were noted, the largest measuring 34 × 16 x 8 mm with a density of 833 Hounsfield units. Both left double-J stents were in place, showing clear signs of encrustation and partial calcification (Figure 2).

With stage 3 CKD, the goal was to prevent the progression of nephrolithiasis-associated nephropathy, with complete stone clearance in a single procedure essential to avoid further deterioration. Open surgical nephrolithotomy and ureterolithotomy were preferred over endoscopic approaches due to the extensive stone burden (342.26 cm^3^ in the kidney and 7.14 cm^3^ in the ureter). Percutaneous nephrolithotomy (PCNL) would have required multiple tracts, increasing the risk of complications, while encrusted stents and multiple ureteral stones would have made it difficult. Ureteroscopy was unfeasible. The left kidney was prioritized due to its relatively better anatomical integrity.

Antibiotic prophylaxis was used. It was adapted to the antibiogram, which showed* P. mirabilis* sensitive to both third-generation cephalosporins (C3G) and aminoglycosides. A combination of ceftriaxone 2 g/day IV and amikacin 15 mg/kg/day IV was administered. Double antibiotic coverage was chosen due to the presence of two long-term JJ stents and a large stone burden, both of which increase the risk of biofilm-related infection and perioperative urosepsis. Prophylaxis was initiated 48 hours before surgery and continued for three days postoperatively (total of five days), in accordance with our department’s protocol for high-risk urological procedures.

**Figure 3:**
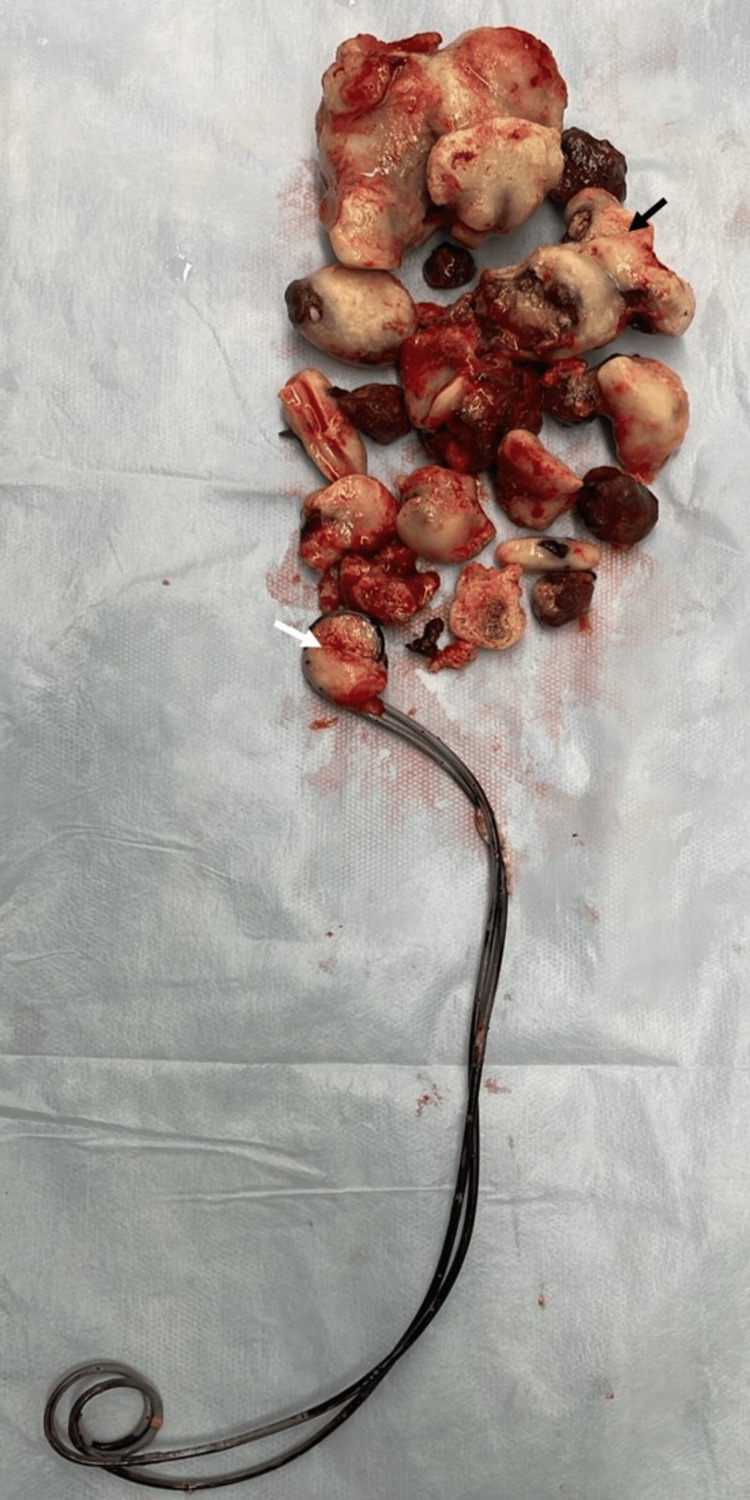
Surgical specimen showing multiple renal calculi (black arrow) and two double-J stents with calcifications at the proximal loop (white arrow).

A left flank lumbotomy incision was performed to provide direct retroperitoneal access to the kidney and upper ureter. After careful dissection and identification of the renal hilum, the renal pelvis was exposed and incised longitudinally. Renal stone extraction proved challenging due to the large size and deep calyceal impaction of the calculi; in addition to the pyelotomy, two separate nephrotomy incisions were required to achieve complete clearance. A proximal ureteral stone mass was palpated manually, and a limited longitudinal ureterotomy was performed to extract the impacted calculi. During the procedure, both previously placed double-J stents were identified and successfully removed (Figure 3). A new double-J stent was placed to maintain ureteral patency, and a nephrostomy tube was inserted for urinary diversion and postoperative drainage. The pyelotomy and nephrotomy incisions were closed in a single layer using 2-0 Vicryl sutures (Ethicon, Inc., Bridgewater, USA) to ensure watertight closure and preserve renal parenchymal integrity. The ureterotomy was closed with interrupted 3-0 Vicryl sutures, ensuring precise, tension-free approximation of the ureteral wall.

The postoperative course was favorable, with modest improvement in renal function (serum creatinine reduced from 2.5 mg/dL to 2.0 mg/dL). The nephrostomy tube was removed on day 7. A follow-up CT confirmed complete stone clearance on the left. One month after surgery, the creatinine improved to 1.8 mg/dL.

After a multidisciplinary uro-nephrology discussion, we decided to proceed with upfront PCNL, aiming to provide the patient with the best chance given his advanced CKD, and to avoid further deterioration of renal function that could lead to the need for dialysis. PCNL is scheduled for the contralateral kidney in six weeks, along with the removal of the left double-J stent.

The spectrophotometric analysis of the stones identified several types according to Daudon et al.'s classification [5]: Type Ia whewellite (caused by diuresis defect and dietary hyperoxaluria), Type IIIc ammonium urate (resulting from hyperuricuria and urinary alkalinization of infectious origin), Type IVc carapatite and struvite (linked to infection with urease-producing bacteria), and Type VIa protein (soft matrix of infectious origin). The necessary medical measures have been implemented following a multidisciplinary discussion.

## Discussion

The specificity of our article lies in the high number and large size of the extracted renal calculi. Nephrolithiasis in CKD patients accelerates renal dysfunction [3] through obstructive uropathy, recurrent infections, and direct parenchymal damage from stones [1]. Large stones cause urinary obstruction, leading to increased intrapelvic pressure, tubular atrophy, and interstitial fibrosis [4]. A rise in renal pelvic pressure above 30 mmHg for more than 24 hours can result in irreversible tubular damage [6]. Hydronephrosis due to obstruction can reduce GFR by up to 50% [3], with a daily decline in renal function estimated at 12% in severe cases [7]. In stage 3 CKD, where the baseline GFR is between 30 and 59 mL/min/1.73m^2^, a further decline below 30 mL/min/1.73m^2^ significantly increases the risk of progression to end-stage renal disease (ESRD) [3], with a five-year ESRD conversion rate exceeding 40% in high-risk patients [1]. Additionally, patients with bilateral nephrolithiasis or recurrent obstructive episodes have a 2.3-fold increased risk of CKD progression compared to those without recurrent obstruction [8].

Urinary tract infections (UTIs) caused by urease-producing bacteria like *P. mirabilis* increase urine pH (often >7.5), promoting the formation of struvite (magnesium ammonium phosphate) and carbonate apatite stones [6]. Urease hydrolyzes urea into ammonia and carbon dioxide, leading to urinary alkalinization and mineral supersaturation, which facilitates crystal formation and rapid stone growth [1]. Struvite stones can develop into staghorn calculi, occupying large portions of the renal pelvis and significantly increasing the risk of chronic infections and obstructive uropathy [8].

These stones serve as a bacterial reservoir due to biofilm formation, which protects pathogens from immune defenses and antibiotic treatment [4]. Persistent infection triggers a prolonged inflammatory response, with elevated levels of pro-inflammatory cytokines such as interleukin-6 (IL-6) and tumor necrosis factor-alpha (TNF-α), leading to chronic tubulointerstitial nephritis [1]. This ongoing inflammation contributes to tubular atrophy and interstitial fibrosis, resulting in progressive renal function [8]. Infectious nephrolithiasis can cause a 40-50% reduction in kidney function within five years if left untreated [4].

Furthermore, patients with recurrent struvite stones have a 1.8- to 2.5-fold increased risk of progressing to ESRD compared to those with non-infectious stones [9]. Chronic colonization by urease-producing bacteria is associated with a 25-30% risk of recurrent urosepsis, further exacerbating renal damage [10].

Neglected ureteral stents and untreated ureteral stenosis significantly impair renal function, with encrustation occurring in 30-50% of long-term cases [9], rising to over 75% after six months [10]. This leads to secondary obstruction, hydronephrosis, and a 1.5- to 2.5-fold increased risk of acute kidney injury (AKI), along with a 10-20% reduction in kidney function [11]. In severe cases, complete ureteral obstruction can increase renal pelvis pressure, further impairing glomerular filtration [10]. Untreated encrusted stents can lead to complications such as pyonephrosis and renal abscesses in up to 20% of cases, accelerating CKD progression [12]. Proper follow-up is essential to prevent irreversible damage [11].

Surgical interventions play a crucial role in preserving renal function in patients with nephrolithiasis and CKD [2]. Postoperative outcomes show a reduction in serum creatinine levels by 0.2-0.5 mg/dL and a 10-20% increase in GFR, depending on preexisting renal damage and stone burden [13]. Early stone removal significantly decreases the risk of recurrent UTIs by up to 40% and reduces the progression to ESRD by nearly 50%, particularly in patients with obstructive uropathy [14].

A multidisciplinary approach is essential for long-term disease management [2]. Alkalinizing agents, such as potassium citrate and sodium bicarbonate, lower urinary acidity and reduce the recurrence of uric acid and calcium oxalate stones by 30-50% [4]. Dietary modifications, including increased fluid intake and reduced oxalate and purine consumption, help control hyperoxaluria and hyperuricemia, decreasing stone formation by 40-60% [13]. Long-term antibiotic prophylaxis is necessary in select cases, reducing the recurrence of infection-related stones by 50-70% and inhibiting the activity of urease-producing bacteria responsible for struvite formation [14].

Spectrophotometric stone analysis is crucial for guiding personalized treatment [2]. Struvite stones, found in 10-15% of recurrent stone formers, are strongly associated with chronic infections and can lead to a 1.5- to 2.5-fold increase in renal function decline if not managed effectively [4]. Uric acid stones, which account for 10-20% of all nephrolithiasis cases, contribute significantly to obstructive uropathy and CKD progression, particularly in patients with metabolic syndrome and insulin resistance [8]. Protein matrix stones (Type VIa), present in 15-20% of patients with recurrent UTIs, require targeted antimicrobial therapy due to their soft, biofilm-rich composition, which promotes bacterial persistence and reinfection [14].

## Conclusions

Timely intervention and regular follow-up are essential to prevent CKD progression in patients with recurrent nephrolithiasis. A multidisciplinary approach, combining surgical intervention, appropriate use of medication based on urine pH, and antibiotics, can improve renal function and reduce infection risks. While alkalinizing agents may be useful in certain cases, it is important to consider that phosphate lithiasis often forms in an alkaline environment, and in such cases, urine acidification may be necessary for optimal management. Spectrophotometric analysis of stones helps personalize treatment, ensuring targeted therapy and early stone clearance. Managing encrusted stents and removing stones early can significantly preserve kidney function and prevent further renal deterioration. Continuous monitoring is critical to ensure long-term renal health and prevent complications in these patients.
